# Electrical percolation in extrinsically conducting, poly(ε-decalactone) composite neural interface materials

**DOI:** 10.1038/s41598-020-80361-7

**Published:** 2021-01-14

**Authors:** Katarzyna Krukiewicz, James Britton, Daria Więcławska, Małgorzata Skorupa, Jorge Fernandez, Jose-Ramon Sarasua, Manus J. P. Biggs

**Affiliations:** 1 Centre for Research in Medical Devices, National University of Ireland, Newcastle Road, Galway, H91 W2TY Ireland; 2 Department of Physical Chemistry and Technology of Polymers, Silesian University of Technology, M.Strzody 9, 44-100 Gliwice, Poland; 3 Department of Mining-Metallurgy Engineering and Materials Science, School of Engineering, POLYMAT, University of the Basque Country (UPV/EHU), Alameda de Urquijo s/n, 48013 Bilbao, Spain; 4 Polimerbio, S.L, Paseo Mikeletegi 83, 20009 Donostia-San Sebastian, Spain

**Keywords:** Neuroscience, Neurology, Chemistry, Materials science

## Abstract

By providing a bidirectional communication channel between neural tissues and a biomedical device, it is envisaged that neural interfaces will be fundamental in the future diagnosis and treatment of neurological disorders. Due to the mechanical mismatch between neural tissue and metallic neural electrodes, soft electrically conducting materials are of great benefit in promoting chronic device functionality. In this study, carbon nanotubes (CNT), silver nanowires (AgNW) and poly(hydroxymethyl 3,4-ethylenedioxythiophene) microspheres (MSP) were employed as conducting fillers within a poly(ε-decalactone) (EDL) matrix, to form a soft and electrically conducting composite. The effect of a filler type on the electrical percolation threshold, and composite biocompatibility was investigated in vitro. EDL-based composites exhibited favourable electrochemical characteristics: EDL/CNT—the lowest film resistance (1.2 ± 0.3 kΩ), EDL/AgNW—the highest charge storage capacity (10.7 ± 0.3 mC cm^− 2^), and EDL/MSP—the highest interphase capacitance (1478.4 ± 92.4 µF cm^−2^). All investigated composite surfaces were found to be biocompatible, and to reduce the presence of reactive astrocytes relative to control electrodes. The results of this work clearly demonstrated the ability of high aspect ratio structures to form an extended percolation network within a polyester matrix, resulting in the formulation of composites with advantageous mechanical, electrochemical and biocompatibility properties.

## Introduction

Neural interfaces typically consist of an array of planar or micro-wire electrodes, implanted onto or into tissues of the central or peripheral nervous system. By providing a bidirectional communication channel between neural tissues and a biomedical device, it is envisaged that neural interfaces will be fundamental in the future diagnosis and treatment of neurological disorders^1^. Traditionally, neural interfaces are made of noble metals, particularly platinum and gold, combining high electrical conductivity with moderate biocompatibility. Nevertheless, due to the mechanical mismatch between the stiff metal surface and excitable tissues, implantable interfacing electrodes illicit an inflammatory response which restricts chronic functionality in vivo^2^. Ongoing efforts to mitigate this problem include employing soft biocompatible polymers with favorable electrical properties as biointerfacing electrodes^3^. In particular, hydrogel formulations including poly(ethylene glycol)^4^, and poly(*N*-isopropylacrylamide) microgels^5^ have been explored extensively as neuroelectrode coatings. Even though these materials are known for their biomimetic mechanical properties, their ability to enhance neuronal survival, and to improve tissue responses at the peri-electrode interface, they are not intrinsically electrically conducting. A further promising group of compounds that is devoid of this drawback and have been explored extensively as neural electrode coatings comprises intrinsically conducting polymers^6^. Although possessing high conductivity and good biocompatibility, conducting polymers are associated with electromechanical instability and increased mechanical stiffness relative to hydrogel formulations^7^.

To avail of their outstanding biocompatibility, non-conducting elastomeric biomaterials can be used as matrices for conducting fillers to produce both conducting and biocompatible composite coatings, which negate the negative effects of the fillers on cell viability and proliferation^8^. So far, several reports have described the design of composite materials of polyurethane and carbon nanotubes^9^, polydimethylsiloxane and carbon nanotubes^10^, polylactide and polyaniline^11^, or more sophisticated, three-composite materials made of carbon nanotubes, polypyrrole and poly(ethylene glycol) diacrylate polyacrylamide^12^. In our studies, an aliphatic polyester was chosen as a biocompatible matrix. Poly(ε-decalactone) (EDL), is a soft elastomeric material possessing high biocompatibility, desirable mechanical properties, and is derived from an environmentally sustainable synthesis process^13^. To endow the polymer with electrical conducting properties, the polymer matrix was loaded with selected fillers differing in size and shape, namely carbon nanotubes (CNT), silver nanowires (AgNW) and conducting polymer microspheres (MSP). The selection of fillers was based on their conductivity as well as the ability to interact with neurons. As described by Sorkin et al.^14^, neural cells are able to directly attach to CNT, and the entanglement of cellular processes can be easily observed on CNT-modified surfaces. Indeed, curly nature of CNT network was found to improve neuronal adhesion, and enhance interactions between CNT and neural cells. The similar observation was made for conducting polymers, particularly PEDOT, which were found to increase the action potentials of PC12 cells as a response to external electrical stimulation^15^, as well as to stimulate retinal neurons at low voltage amplitudes and low charge densities^16^. In our previous study^13^, we described neural cell stimulation experiments confirming that AgNW are able to transfer charges suitable for cellular depolarization, increasing stimulation efficiency when compared with a control electrode. To determine the minimum amount of a filler required to provide a favourable conductive profile to the polyester matrix, electrochemical impedance spectra (EIS) and cyclic voltammograms (CV) of EDL-based composites were recorded, and the corresponding values of film resistance, capacitance (C) and charge storage capacity (CSC) were extracted. The morphology of the material-tissue interface was examined using scanning electron microscopy to visualize percolation network of filler particles. Finally, the biocompatibility of the composites was assessed in vitro using ventral mesencephalon (VM) cells derived from embryonic rat brain tissue.

## Results

### Surface morphology

**Figure 1:**
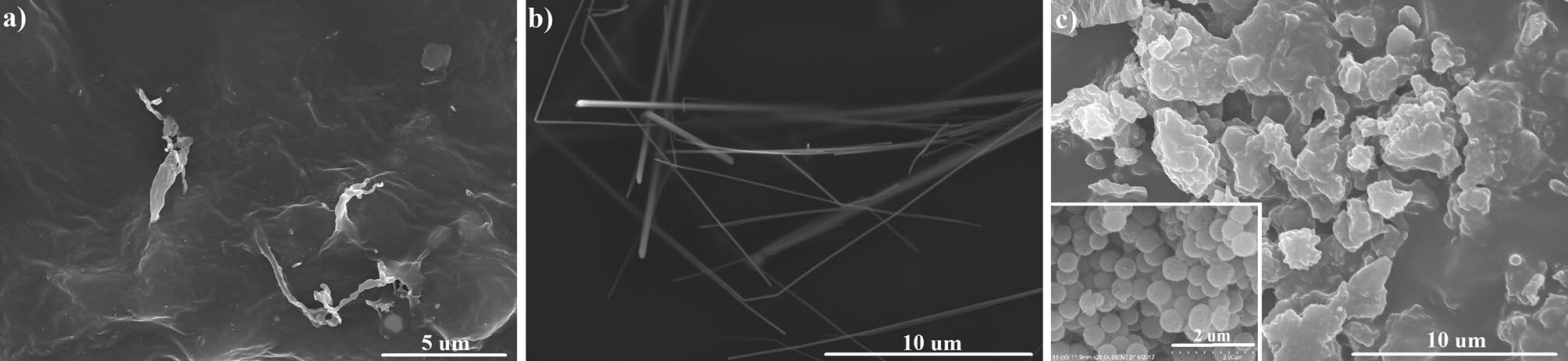
Surface characterization of EDL-based composites. SEM micrographs of EDL/CNT (**a**), EDL/AgNW (**b**) and EDL/MSP (**c**), the latter with the inset showing P(EDOT-OH) microspheres after synthesis.

The presence of each filler on the surface morphology of EDL-based composites was assessed by SEM imaging, as shown in Fig. 1. In the case of EDL/CNT nanocomposites (Fig. 1a), the surface morphology was characterised by the presence of non-uniform elongated protrusions with a length between 1.5 μm and 5 μm, and a width between 50 to 400 nm, significantly larger than the dimensions of the filler CNT (outer diameter of 10 nm, inner diameter of 4.5 nm, length of 3–6 μm). The presence of surface filler particles was also evident in EDL/AgNW nanocomposite materials (Fig. 1b). Interestingly, AgNW nanowires protruded from the composite surface, with a fraction of these demonstrating deformation. The morphology of EDL/MSP composites (Fig. 1c) was characterised by a rough surface of nodules possessing an average diameter of 2 µm, four times higher than the diameter of individual MSP (0.5 µm, Fig. 1c-inset).

### Electrochemical characterisation

**Figure 2:**
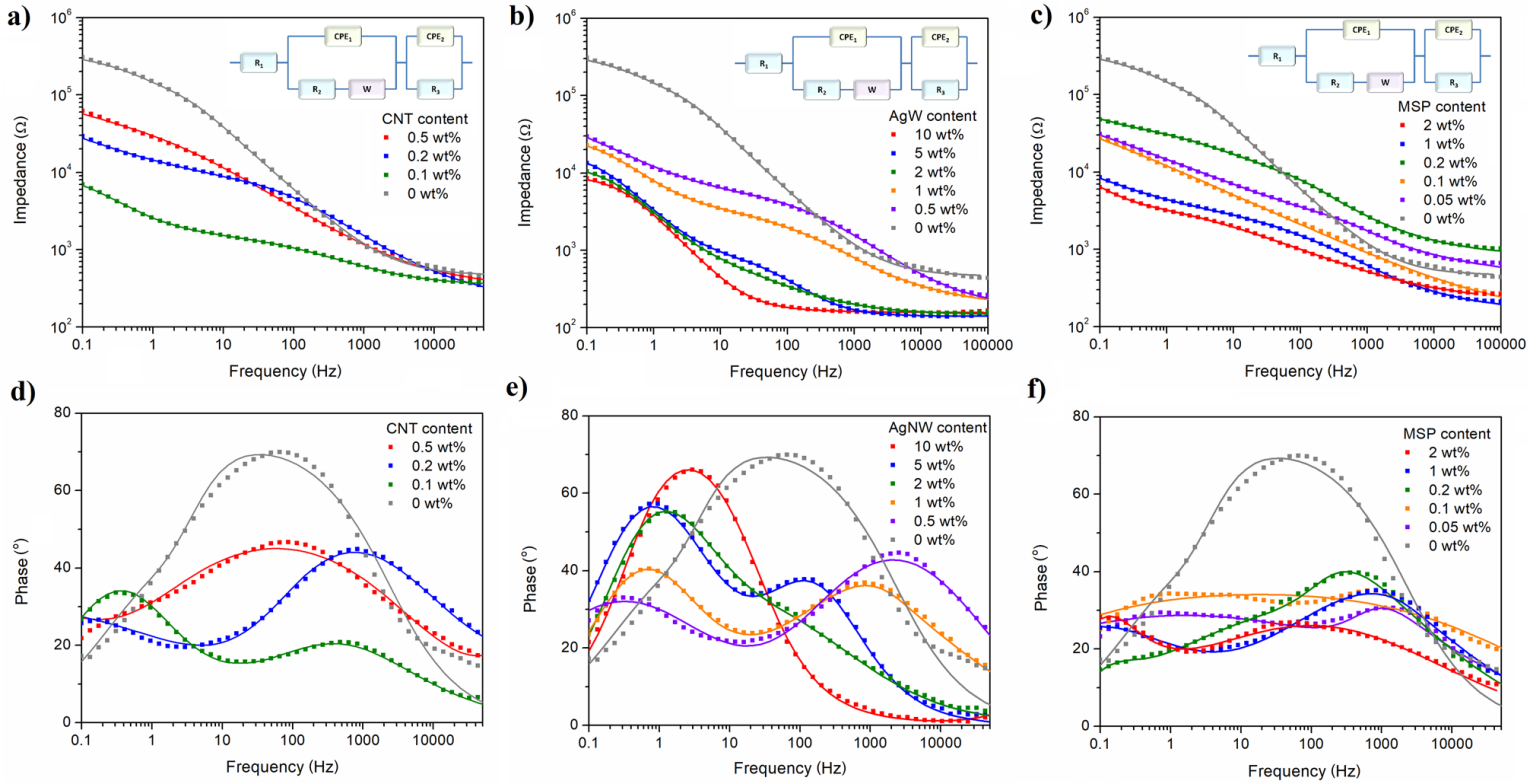
Electrochemical impedance spectroscopy study of EDL-based composites. Bode plots showing the frequency-dependent behaviour of the impedance modulus of EDL/CNT (**a**), EDL/AgNW (**b**) and EDL/MSP (**c**), and phase angle of EDL/CNT (**d**), EDL/AgNW (**e**) and EDL/MSP (**f**) composites; dots represent experimental data, while lines represent simulated results; equivalent circuit models used for the fitting of EIS data are presented as the insets. EIS data were collected in 0.1 M KCl solution, in the frequency range from 0.1 Hz to 100 kHz, with an AC amplitude of 40 mV (vs. Ag/AgCl) and a DC potential equal to 0 V (vs. Ag/AgCl). EIS simulations were performed by means of EIS Spectrum Analyzer 1.0 software (http://www.abc.chemistry.bsu.by/vi/analyser/).

**Figure 3:**
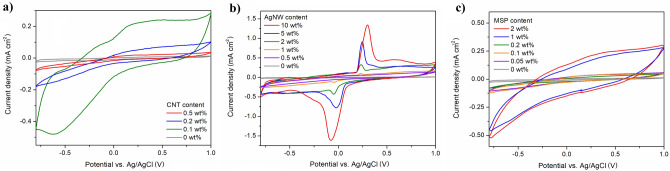
Electrochemical behaviour of EDL-based composites. Cyclic voltammetric curves of EDL/CNT (**a**), EDL/AgNW (**b**) and EDL/MSP (**c**) composites collected in 0.1 M KCl solution within the potential range from − 0.8 to 1.0 V (vs. Ag/AgCl) at 100 mV s^−1^.

**Figure 4:**
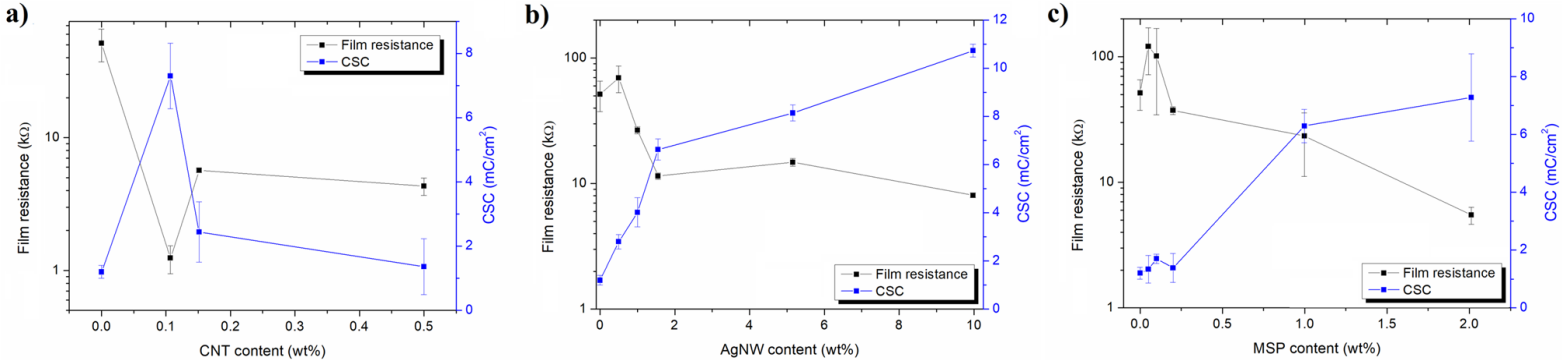
Electrochemical percolation threshold for EDL-based composites. Film resistance/charge storage capacity (CSC) plots of EDL/CNT (**a**), EDL/AgNW (**b**) and EDL/MSP (**c**) composites; n = 3. Film resistance values were determined using an equivalent circuit modelling of EIS data (R_3_), while CSC values were calculated from CV curves.

Electrochemical behaviour of EDL/CNT, EDL/AgNW and EDL/MSP composites was assessed by electrochemical impedance spectroscopy (Fig. 2) and cyclic voltammetry (Fig. 3), and the results were used to evaluate the mean film resistance, capacitance and charge storage capacity (CSC) of each material (Fig. 4). For both EDL/AgNW and EDL/MSP, an increase in a filler content led to a decrease in the impedance of the material over the whole frequency range employed (Fig. 2b,c). In contrast, EDL/CNT formulations (Fig. 2a), demonstrated the lowest impedance profile with a 0.1 wt% CNT content. Similarly, the phase profile of EDL/CNT formulations (Fig. 2d) containing 0.1 wt% CNT content differed significantly from the phase profile of pristine EDL, showing two distinct phase angle peaks (0.4 Hz ad 450 Hz), in opposition to a single phase angle peak at the frequency of ~ 70 Hz observed for pristine EDL. A similar two-peak phase angle profile was observed for EDL/MSP (Fig. 2f), with phase peaks noted at the frequencies of 0.15 Hz and 75 Hz. In the case of EDL/AgNW (Fig. 2e), a two-peak phase angle profile was observed for composite formulations with 0.5–5 wt% AgNW content. When the AgNW content was increased to 10 wt%, a single phase peak could be observed at the frequency of 3 Hz.

An increase in the area under the CV curve with no defined indication of reduction/oxidation peaks was observed for EDL/CNT (Fig. 3a), particularly for a composite formulated with 0.1 wt% CNT content. Conversely, the CV curves of EDL/AgNW nanocomposites (Fig. 3b) were characterized by a distinctive reduction–oxidation system at the potentials of − 0.05 V (reduction of silver) and 0.25 V (oxidation of silver), appearing for composites with AgNW contents above 0.5 wt%. With an increase in AgNW content, an increase in the peak current was observed, accompanied by a small increase in peak separation (from 270 mV for EDL/AgNW 1 wt% to 380 mV for EDL/AgNW 10 wt%). Conversely, an increase in the area under the CV curve with no defined indication of reduction/oxidation peaks was observed for EDL/MSP (Fig. 3c), starting from an MSP content of 1 wt%.

By integrating CV curves in the time domain, the charge storage capacities (CSC) of EDL composites were calculated and compared with capacitances and resistances determined from the equivalent circuit modelling of the EIS data (Fig. 4a–c). One of the most common models is a Randles circuit, which is frequently used to simulate electrode–electrolyte interface impedance of coated electrodes^17^. For the purpose of modelling, a modified Randles circuit was used, including a solution resistance (R_1_), charge transfer resistance (R_2_), constant phase element modeling the non-faradaic processes (CPE_1_), Warburg element representing the finite length diffusion of faradaic current (W), resistance to charge conduction in the solid electrolyte interphase layer (film resistance, R_3_) and charge capacitance of interphase layer (CPE_2_)^18^, with the quality of fitting demonstrated by the fitted curves in the Bode plots (Fig. 2a–f) and low percentage deviation between experimental data and fitted values (Table 1). Among all samples, the highest value of CSC (10.7 ± 0.3 mC cm^−2^) was noted for EDL/AgNW (10 wt%), and the lowest value of film resistance (1.2 ± 0.3 kΩ) was noted for EDL/CNT (0.1 wt%). Further increases in the CNT content did not increase the CSC or decrease the film resistance of EDL/CNT nanocomposites, but had a deteriorating effect on their electrochemical behaviour. Conversely, for both EDL/AgNW and EDL/MSP composite materials, increases in the filler content led to enhancement of the electrochemical properties. Although the percolation threshold for EDL/AgNW was observed at 2 wt% content (CSC of 6.6 ± 0.4 mC cm^−2^, film resistance of 11.5 ± 0.6 kΩ), further increases in the AgNW content increased the CSC to a maximum of 10.7 ± 0.3 mC cm^−2^ and decreased the film resistance to a minimum of 8.1 ± 0.3 kΩ for EDL/AgNW (10 wt%). For EDL/MSP composites, the percolation threshold was also observed at 2 wt% MSP content, resulting in a CSC of 7.3 ± 1.5 mC cm^−2^ and a film resistance of 5.5 ± 0.8 kΩ.

**Table 1:** Representative set of electrical parameters derived from an equivalent circuit modelling of EIS data for pristine EDL, EDL/CNT 0.1 wt%, EDL/AgNW 2 wt% and EDL/MSP 2 wt%.

Circuit element	EDL	EDL/CNT 0.1 wt%	EDL/AgNW 2 wt%	EDL/MSP 2 wt%
Solution resistance (R_1_), Ω	465	338	146	208
Charge transfer resistance (R_2_), Ω	296,000	2450	643	4417
Resistance in the solid electrolyte interphase layer (R_3_), Ω	51,464	1242	11,483	5483
CPE parameter (P_1_), µS s^n^	2.1	161.1	134.0	63.8
CPE parameter (n_1_)	0.733	0.751	0.533	0.432
Capacitance of non-faradaic processes (C_1_), µF	1.8	119.0	15.6	12.2
CPE parameter (P_2_), µS s^n^	0.9	20.8	67.8	416.4
CPE parameter (n_2_)	1.000	0.558	0.884	0.993
Capacitance of interphase layer (C_2_), µF	0.9	1.2	65.6	418.4
Warburg coefficient (W_sr1_), pΩ s^−1/2^	0.19	0.25	7.41	0.97
Warburg parameter (W_sc1_)	0.7	7.9	8.7	8.7
% deviation (χ)	9.1	1.8	2.9	1.8

The comparison of electrical parameters derived from an equivalent circuit modelling of EIS data for representative EDL-based composites (Table 1) further highlighted the effect of the presence of particular conducting fillers on the electrochemical properties of fabricated materials. Charge transfer resistance was found to dramatically decrease when EDL matrix (296 kΩ) was filled with either CNT (2 450 Ω), AgNW (643 Ω) or MSP (4 417 Ω). The similar behaviour was observed for the resistance in the solid electrolyte interphase layer (film resistance, R_3_), which was found to decrease from 5 to 50 times for EDL/AgNW and EDL/CNT, respectively. The improvement was also observed in the capacitive behaviour of the composites, indicating EDL/CNT as having the highest capacitance of non-faradaic processes (119.0 µF), and EDL/MSP as exhibiting the highest interphase capacitance (418.4 µF). A high variation of Warburg parameters, particularly W_sc_, between pristine EDL and EDL-based composites indicated limited diffusion of faradaic current observed for pristine polymer, and the similar diffusive behaviour of all EDL-based composite materials.

For the subsequent biological characterization, EDL-based composites with the filler content above percolation threshold were chosen, exhibiting the electrical properties summarized in Table 2.

**Table 2:** Summary of electrical parameters of pristine EDL, EDL/CNT 0.1 wt%, EDL/AgNW 2 wt% and EDL/MSP 2 wt%.

Parameter	EDL	EDL/CNT 0.1 wt%	EDL/AgNW 2 wt%	EDL/MSP 2 wt%
Film resistance, kΩ	51.4 ± 14.1	1.2 ± 0.3	11.5 ± 0.6	5.5 ± 0.8
Charge storage capacity (CSC), mC cm^−2^	1.2 ± 0.2	6.3 ± 1.0	6.6 ± 0.4	7.3 ± 1.5
Areal capacitance of non-faradaic processes, µF cm^−2^	6.4 ± 1,4	420.5 ± 12.6	55.1 ± 1.7	43.1 ± 2.6
Areal capacitance of interphase layer (C_2_), µF cm^−2^	3.2 ± 0.7	4.2 ± 0.2	231.8 ± 6.3	1478.4 ± 92.4

### In vitro biocompatibility

**Figure 5:**
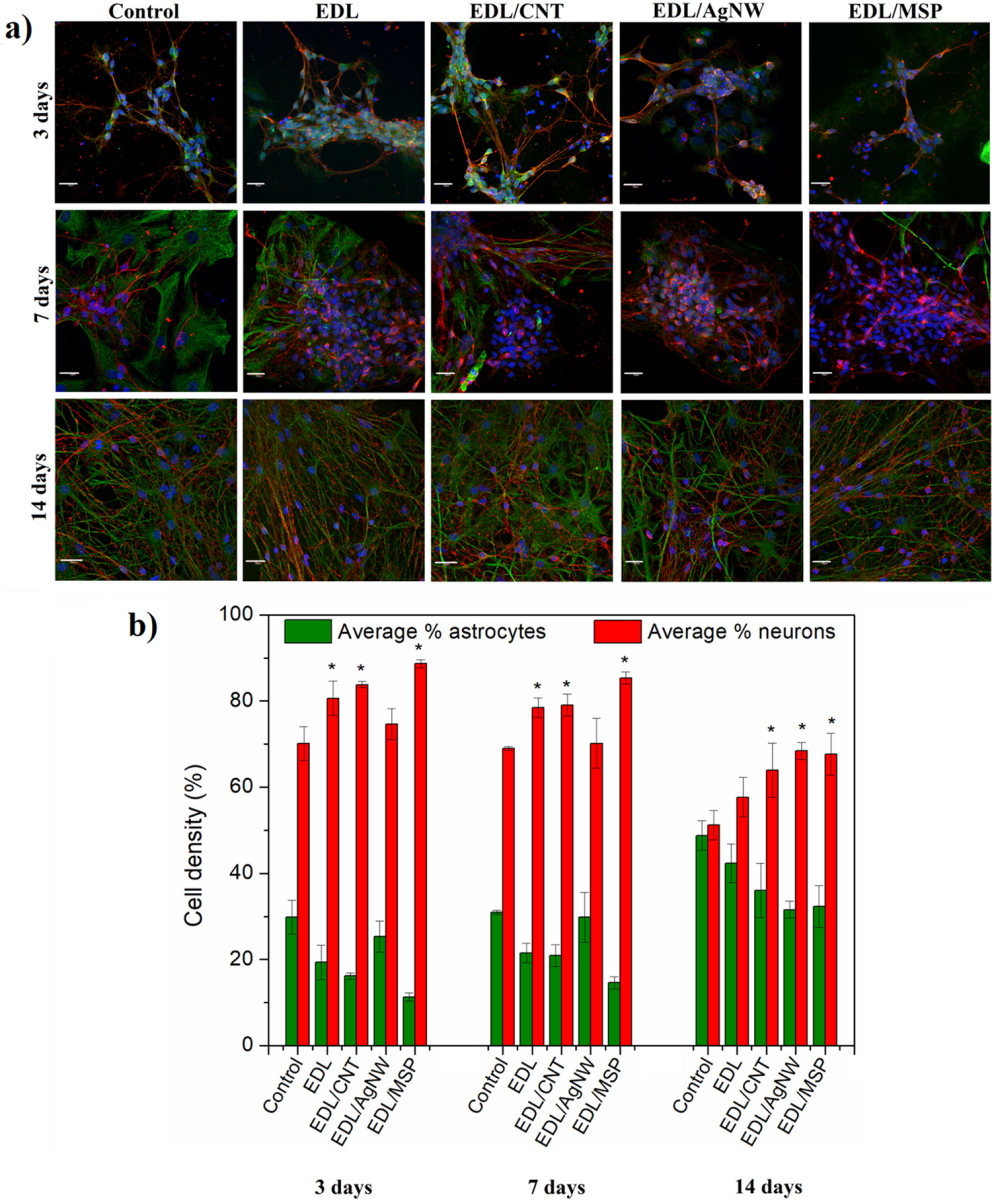
Biocompatibility of EDL-based composites. Fluorescent images of primary ventral mesencephalic mixed cell population cultured for 3, 7 and 14 days on a control substrate (bare Pt-coated glass slide), pristine EDL, EDL/CNT, EDL/AgNW and EDL/MSP; neurons are visualized by anti β-tubulin III (red), astrocyte cells by anti-GFAP (green) and nuclei by DAPI (blue); the scale bar represents 20 μm (**a**). Cell density (%) analysis of astrocyte and neuron presence on each of the experimental and control group (**b**); n = 3, * = p < 0.05.

The cytocompatibility of EDL-based composites was assessed with respect to a mixed neural population obtained from ventral mesencephalon (VM) of embryonic Sprague–Dawley rats. VM cells were cultured on the surface of all experimental composite and control materials for 3, 7 and 14 days in vitro, and were visualized by dual immuno-staining to distinguish neurons and astrocytes (Fig. 5a). Further quantification was conducted to extract the percentage of neurons and astrocytes present on each of the experimental composites and control substrates (Pt and EDL coated glass slides) (Fig. 5b). At each time point, EDL-based coatings were observed to favour the presence of neurons over astrocytes with respect to Pt control substrates. After 3 days, the percentage of neurons was highest on EDL (80.6 ± 4.0%), EDL/CNT (83.8 ± 0.7%) and EDL/MSP (88.7 ± 1.0%), a trend maintained at all experimental time points. Although by day 14 the percentage of cells cultured on pristine EDL coatings (57.7 ± 4.5%) was not found to differ significantly from cells cultured on Pt control substrates (51.2 ± 3.4%), all EDL-based composites were observed to lead to an increase in the presence of neurons relative to astrocyte cells, achieving a neuron distribution percentage of 64.0 ± 6.2%, 68.4 ± 2.0% and 67.7 ± 4.9% for EDL/CNT, EDL/AgNW and EDL/MSP, respectively.

## Discussion

Materials employed as neural interfaces should possess high electrical conductivity and cytocompatibility with biomimetic mechanical properties. To meet these requirements, we described a group of composite materials consisting of poly(ε-decalactone) (EDL) as a soft and biocompatible polymer matrix, and electrically conducting fillers, namely multi-walled carbon nanotubes (CNT), silver nanowires (AgNW) and poly(hydroxymethyl 3,4-ethylenedioxythiophene) microspheres (MSP). EDL, a polymer derived from a commercially available, renewable ε-decalactone, has been recently described as an effective neural interface material, possessing favourable mechanical properties and enhancing neurite outgrowth in vitro^13^. Due to its viscous nature, EDL can be easily used in simple coating processes, such as dip- and spin-coating. Critically, however, EDL is not an intrinsically electrically conducting polymer. To promote conductivity, a highly conductive filler must be added to the EDL polymer matrix, to form a three dimensional network of filler particles, known as a percolation network. At a particular concentration of the filler, a dramatic increase in conductivity and capacitance of a composite is observed. This so-called percolation threshold is highly dependent on the aspect ratio of the filler particles, and is usually observed between 0.05 and 10 wt%^19^.

Among the three fillers investigated in this study, CNT are the most frequently used for neural applications, mainly because of their extraordinary strength, electrical conductivity and chemical stability^20^. Nanocomposites of polymers with CNT exhibit high charge injection limits and low impedance^20^, and promote cell viability^21^, however the electrical percolation threshold of CNT nanocomposites has been reported to vary considerably. Specifically, a percolation threshold was obtained at 0.5 wt% CNT content with a polyhydroxyalkanoate matrix^21^, 1–5 wt% CNT content with a chitin matrix^22^, and 18 wt% CNT content with a polyethyleneimine matrix^23^. In this study, we noted a low percolation threshold of 0.1 wt% for CNT characterized by an aspect ratio (length-to-width) of 450 and conductivity of 35 S cm^−1^ ^24^. Surprisingly, when the content of CNT was further increased, an increase in the EDL nanocomposite film resistance and a decrease in CSC were observed, suggesting that above this CNT content interactions between EDL and CNT, probably derived from a viscous nature of EDL, facilitate the formation of CNT agglomerates, suppressing the development of a robust percolation network. With a CNT content of 0.1 wt%, the value of film resistance (1.2 ± 0.3 kΩ) was the lowest among all composites investigated in this study, irrespective of the amount of filler content. EDL/CNT composites were also found to be highly capacitive, particularly in terms of the capacitance of non-faradaic processes (420 µF cm^−2^), associated with the redistribution of charges at the surface of CNT. Although materials with high capacitances are generally desired in the design of neural electrodes, they are not favourable for rapid electrochemical measurements, since high capacitance limits the response time of the device^25^. To allow the use of fast scan rates, the capacitance of the electrode should be of ∼ 100 μF cm^−2^, which is four times lower than noted for EDL/CNT.

EDL composites formulated from a AgNW filler (aspect ratio of 1500, conductivity of 6.3 × 10^7^ S m^−1^) were found to possess a percolation threshold at 2 wt% content. Although this value was within a range previously identified for similar AgNW composite materials^26^, it was significantly higher than a percolation threshold noted for EDL/CNT composites. It is hypothesised that this increase is related to the filler density: 10.49 g cm^−3^ for Ag and 1.6 g cm^−3^ for CNT. Consequently, the same wt% of each filler should result in a 6.5 × increase in the volume of CNTs in the EDL matrix relative to AgNW, facilitating the formation of a more robust CNT percolation network. Conversely, the substantial length and ductility of the AgNWs facilitated a decrease in film resistance of EDL/AgNW composites with increased AgNW content, up to 8.1 ± 0.3 kΩ. CSC is an important consideration in neuroelectrode fabrication and can be used to predict how much charge can be injected through an electrode during electrical stimulation. Although demonstrating a moderate conductivity, EDL/AgNW nanocomposites exhibited the highest CSC (10.7 ± 0.3 mC cm^−2^) of all investigated composites, as well as other neural electrode materials described in the literature, including PEDOT (~ 7 mC cm^−2^)^27^, and PEDOT-CNT composites (~ 8.6 mC cm^−2^)^28^. Due to the electrochemical behaviour of AgNW, which undergo reduction/oxidation reactions^29^, a substantial amount of charge (linearly dependent on the concentration of AgNW) is transferred between the electrode and AgNW, giving rise to the redox system of peaks clearly observed in the CV curves. Furthermore, the presence of an additional capacitive peak at low frequencies in the phase angle profile of EDL/AgNW composites confirmed the capacitive nature of this material^30^. Therefore, EDL/AgNW was found to exhibit relatively high interphase capacitance (231.8 ± 6.3 µF cm^−2^) together with moderate capacitance of non-faradaic processes (55.1 ± 1.7 µF cm^−2^), behaving similarly as Kevlar/graphene oxide electrodes^31^. The moderate value of capacitance allows to keep the time needed to charge and discharge the electrode low, enabling to use fast scans for neural recording and to minimize background currents^32^. Although pristine conducting polymer electrode coatings exhibit excellent electrochemical properties, EDL composites formulated with P(EDOT-OH) MSP performed similarly as EDL loaded with CNT or AgNW nanocomposites. The film resistance of EDL/MSP composites (5.5 ± 0.8 kΩ) was lower than that observed for the EDL/AgNW nanocomposites, but CSC of 7.3 ± 1.5 mC cm^−2^ was noted to be lower than CSC of EDL/AgNW, and comparable with the CSC value of EDL/CNT. Interestingly, EDL/MSP were found to exhibit the highest areal interphase capacitance (1478.4 ± 92.4 µF cm^−2^), which should be associated with the processes of charging/discharging of the electrode–electrolyte double layer, as indicated by the presence of a CV curve typical for PEDOT^33^. High interphase capacitance should be derived from the favourable geometry of MSP (aspect ratio of 1), which allows for maximizing the surface-area-to-volume ratio.

The results of biological investigations confirmed that EDL-based composites promoted neuron and astrocyte adhesion and viability, irrespective of the filler type. At early time points (3 days), pristine EDL and EDL/CNT and EDL/MSP composites exhibited an increased presence of neurons relative to a control Pt surface. A similar trend was observed at day 7. Following 14 days in culture, EDL-coated surfaces possessed a similar neuron/astrocyte coverage to control Pt substrates. Conversely, all investigated composites significantly increased the presence of neurons at 14 days of culture. Astrocytes are supporting cells that are present in a healthy neural population and it is accepted that they play a role in the modulation of neural signalling^34^. Moreover, astrocytes are known to play an active role in gliosis^35^, a critical mediator of neural interface failure in vivo. Here, EDL-based composites were found to reduce astrocyte surface coverage while maintaining a high neuronal coverage, indicating that they may enhance neuron-electrode coupling and suppress the formation of glial scarring in vivo.

## Conclusions

In this study, the effects of carbon nanotubes (CNT), silver nanowires (AgNW) and P(EDOT-OH) microspheres (MSP) conducting fillers on the electrical percolation threshold and performance of a neural interface material based on a biocompatible poly(ε-decalactone) matrix were investigated. Due to the geometrical shape of fillers, ranging from spherical (MSP) to high aspect ratio (CNT, AgNW) structures, the percolation threshold was found to vary between 0.1 wt% (EDL/CNT) and 2 wt% (EDL/AgNW and EDL/MSP). The most favourable electrical characteristics in terms of low film resistance (1.2 ± 0.3 kΩ) and high CSC (10.7 ± 0.3 mC cm^−2^) was noted for EDL/CNT and EDL/AgNW nanocomposites, respectively. Conversely, the electrical performance of EDL/MSP was in the range of applicability, but due to the high value of interphase capacitance (1478.4 ± 92.4 µF cm^−2^) this composite seemed not to be favourable for rapid electrochemical measurements. All investigated composites were found to be cytocompatible, and reduced the surface astrocyte load in vitro, which may enhance neuron-electrode coupling and suppress the formation of a peri-interface glial scar in vivo. The results of our work clearly demonstrated the potency of selected fillers, CNT and AgNW, to form an extended percolation network within the matrix of an environmentally sustainable polyester, EDL, resulting in advantageous electrochemical properties while maintaining a high cytocompatibility. In conclusion, EDL-based conducting composites were shown to serve as advantageous neural interface materials in vitro.

## Methods

### Synthesis and characterization of poly(ε-decalactone)

Poly(ε-decalactone) (EDL) was synthesized according to a method described previously^13^. In short, after purging ε-decalactone (> 99%, Sigma Aldrich) with nitrogen, it was mixed with the catalyst (Ph_3_Bi, Gelest) at 100:1 monomer/catalyst molar ratio and stirred for 7 days. After this time, the product was dissolved in chloroform, precipitated, dried at room temperature and heated to ensure the complete elimination of any remaining solvent.

### Fabrication of P(EDOT-OH) microspheres

Poly(hydroxymethyl 3,4-ethylenedioxythiophene) hollow microspheres (MSP) were fabricated using a sacrificial template chemical polymerisation process^36^. In brief, EDOT-OH was dissociated in a sonication bath at a frequency of 35 kHz for 30 min. Following this, polystyrene (PS) beads with an average diameter of 500 nm (Spherotech inc., CHI, USA) were added to the EDOT-OH solution to a final concentration of 2.5 mg ml^−1^. The mixture was allowed to homogenise using a magnetic stirrer for 30 min before ammonium persulphate was added to initiate polymerisation of EDOT-OH around PS bead templates. The solution was then mixed for 16 h at room temperature before the addition of excess methanol ceased the reaction. Following this, the solution was centrifuged (Thermo Scientific HERAEUS FRESCO 17 Centrifuge) at 13,000 rpm for 15 min to remove excess neutralised solution, and washed three times with deionized water by resuspension and repetitive centrifugation. To dissolve the internal polystyrene template, MSP were suspended in tetrahydrofuran, THF (Sigma Aldrich) for 15 min followed by repetitive washing in deionized water as described earlier.

### Fabrication of composites

100 mg of EDL and 1 ml of THF (Sigma Aldrich) were mixed, heated to 40 °C and stirred until dissolution. Then, filler particles: silver nanowires (ACS Material, average diameter 100 nm, average length 100–200 μm, silver purity ~ 99.5%, 20 mg ml^−1^), multi-walled carbon nanotubes (Sigma Aldrich, 4.5 nm inner diameter, 10 nm outer diameter, 3–6 µm length) or P(EDOT-OH) microspheres (0.5 µm diameter), respectively, were added to the EDL/THF solution, to give a dispersion with the filler weight ratio from 0.05 wt% to 10 wt%. The dispersion was then mixed via ultrasonication (SONICS Sonicator, 40% amplitude, 3.5 MJ, pulsing: 5 s on and 2 s off) for 1 min. Prior to the deposition of a coating, microscopic glass slides (2.5 × 2.5 × 0.1 cm) were sputter-coated (Emitech K650XT Sputter Coater, 25 mA, 1 × 10^− 3^ mbar, 180 s) with a thin layer of Pt (approx. 5 nm). Pristine EDL, EDL/CNT, EDL/AgNW and EDL/MSP coatings were deposited through a spin coating process (Laurell Technologies Spin Coater) of 0.2 ml of relevant dispersion for 20 s with a speed of 3000 rpm. After this, coated glass slides were dried for 1 h at 50 °C to evaporate the residual THF. Homogeneous and uniform coating (5.8 ± 0.8 μm) was formed when EDL concentration was equal to 10 wt%.

### Characterization of composites

The surface morphology of EDL/CNT, EDL/AgNW and EDL/MSP coatings was analysed with scanning electron microscopy (Hitachi S-4700 Scanning Electron Microscope, 15 kV). Electrochemical characterization of pristine EDL, EDL/CNT, EDL/AgNW and EDL/MSP coatings was performed by means of a PARSTAT 2273 potentiostat in a three-electrode set-up, comprising Ag/AgCl as a reference electrode, glassy carbon rod as an auxiliary electrode and coated glass slides as a working electrode (exposed area of 0.283 cm^2^). Charge storage capacity (CSC) was calculated from the area below cyclic voltammetric (CV) curves collected in 0.1 M KCl solution, within the potential range from − 0.8 to 1.0 V (vs. Ag/AgCl) at 100 mV s^−1^. Electrical parameters of coatings were calculated basing on the equivalent circuit modelling of electrochemical impedance spectra (EIS) collected in 0.1 M KCl solution with frequencies ranging from 100 MHz to 100 kHz, an AC amplitude of 40 mV (vs. Ag/AgCl) and a DC potential equal to 0 V (vs. Ag/AgCl). The experimental EIS data were fitted to a selected equivalent circuit model (modified Randles circuit) with the use of an EIS Spectrum Analyzer 1.0 software (http://www.abc.chemistry.bsu.by/vi/analyser/)^37^. A Powell algorithm was used to fit the data, and the fitting procedure was continued until reaching a satisfactory goodness of fit (the deviation of experimental and fitted data < 5%, with the exception of pristine EDL which had strongly resistive behaviour). In the course of fitting, the following parameters were assessed: a solution resistance (R_1_), charge transfer resistance (R_2_), constant phase element modeling the non-faradaic processes (CPE_1_), Warburg element representing the finite length diffusion of faradaic currents (W), resistance to charge conduction in the solid electrolyte interphase layer (film resistance, R_3_) and charge capacitance of interphase layer (CPE_2_).

Capacitance was calculated basing on the parameters of a constant phase element (CPE) according to the formula:

$$C = \frac{{(P \cdot R)^{{\frac{1}{n}}} }}{R}$$

where *C* is the capacitance (F), *R* is the resistance (Ω), *P* and *n* are CPE parameters.

### In vitro biological characterization

To determine the cytocompatibility of pristine EDL, EDL/CNT, EDL/AgNW and EDL/MSP coatings, a mixed neural population obtained from the ventral mesencephalon of E14 rat embryos was cultured on the surface of coated glass slides for 14 days, according to a previously established protocol^38^. The embryos were obtained by laparotomy from time-mated female Sprague–Dawley rats. All experiments were performed in accordance with the EU guidelines (2010/63/UE) and were approved by the Health Products Regulatory Authority (AE19125/I179) and the National University of Ireland, Galway research ethics committee. The study was carried out in compliance with the ARRIVE guidelines. Every effort was made to minimize animal suffering and to reduce the number of animals used.

Cell cultures were visualized through an indirect double-immunofluorescent labelling method: neurons were stained red (anti-β-tubulin III antibody produced in rabbit, Alexa Fluor 594 goat anti-rabbit IgG (H + L)), astrocytes were stained green (anti-GFAP antibody in mouse, Alexa Fluor 488 goat anti-mouse IgG/IgA/IgM (H + L)), cell nuclei were stained blue (4′,6-diamidino-2-phenylindole, DAPI)^38^. Fluorescent images were collected by an Olympus Fluoview 1000 Confocal Microscope (scan size of 1024 × 1024 at a ratio 1:1 and 60 × magnification). To analyse cell density, the number of nuclei corresponding to neurons and astrocytes was counted in an area of 212 μm × 212 μm in at least 6 random images taken from investigated groups.

Three biological replicates were employed to determine cytocompatibility of all investigated materials. Statistical significance (p < 0.05) was determined by one-way Anova and *t*-test, and the results were presented as the mean of the values ± standard deviation.

## Data Availability

The datasets generated and analysed during the current study are available from the corresponding author on reasonable request.
